# Erythrocytes from patients with ST-elevation myocardial infarction induce cardioprotection through the purinergic P2Y_13_ receptor and nitric oxide signaling

**DOI:** 10.1007/s00395-022-00953-4

**Published:** 2022-09-16

**Authors:** Tong Jiao, Aida Collado, Ali Mahdi, Juliane Jurga, John Tengbom, Nawzad Saleh, Dinos Verouhis, Felix Böhm, Zhichao Zhou, Jiangning Yang, John Pernow

**Affiliations:** 1 Department of Medicine, Division of Cardiology, Karolinska Institutet, Stockholm, Sweden; 2 Department of Cardiology, Karolinska University Hospital, Stockholm, Sweden

**Keywords:** ST-elevation myocardial infarction, Red blood cells, Ischemia–reperfusion injury, ATP, Nitric oxide, Purinergic, Soluble guanylyl cyclase

## Abstract

Red blood cells (RBCs) are suggested to play a role in cardiovascular regulation by exporting nitric oxide (NO) bioactivity and ATP under hypoxia. It remains unknown whether such beneficial effects of RBCs are protective in patients with acute myocardial infarction. We investigated whether RBCs from patients with ST-elevation myocardial infarction (STEMI) protect against myocardial ischemia–reperfusion injury and whether such effect involves NO and purinergic signaling in the RBCs. RBCs from patients with STEMI undergoing primary coronary intervention and healthy controls were administered to isolated rat hearts subjected to global ischemia and reperfusion. Compared to RBCs from healthy controls, RBCs from STEMI patients reduced myocardial infarct size (30 ± 12% RBC healthy vs. 11 ± 5% RBC STEMI patients, *P* < 0.001), improved recovery of left-ventricular developed pressure and dP/dt and reduced left-ventricular end-diastolic pressure in hearts subjected to ischemia–reperfusion. Inhibition of RBC NO synthase with L-NAME or soluble guanylyl cyclase (sGC) with ODQ, and inhibition of cardiac protein kinase G (PKG) abolished the cardioprotective effect. Furthermore, the non-selective purinergic P2 receptor antagonist PPADS but not the P1 receptor antagonist 8PT attenuated the cardioprotection induced by RBCs from STEMI patients. The P2Y_13_ receptor was expressed in RBCs and the cardioprotection was abolished by the P2Y_13_ receptor antagonist MRS2211. By contrast, perfusion with PPADS, L-NAME, or ODQ prior to RBCs administration failed to block the cardioprotection induced by RBCs from STEMI patients. Administration of RBCs from healthy subjects following pre-incubation with an ATP analog reduced infarct size from 20 ± 6 to 7 ± 2% (*P* < 0.001), and this effect was abolished by ODQ and MRS2211. This study demonstrates a novel function of RBCs in STEMI patients providing protection against myocardial ischemia–reperfusion injury through the P2Y_13_ receptor and the NO–sGC–PKG pathway.

**Supplementary Information:**

The online version contains supplementary material available at 10.1007/s00395-022-00953-4.

## Introduction

The red blood cell (RBC) has during recent years gained considerable interest as a regulator of cardiovascular function besides its primary role in the delivery of respiratory gases [7, 27, 36]. It has been proposed that RBCs are involved in the regulation of vascular function, especially by inducing vasodilatation under hypoxic and ischemic conditions via the release of nitric oxide (NO) bioactivity and ATP [11, 16, 34, 38]. This effect of RBCs can be achieved due to their expression of a functionally active endothelial NO synthase (eNOS) and soluble guanylyl cyclase (sGC), the intracellular receptor for NO, to produce NO bioactivity [7, 8, 23]. The important role of RBC eNOS in the regulation of blood pressure was recently demonstrated in an RBC-specific eNOS knockout mouse model [25]. RBCs have also been shown to protect the myocardium in the setting of ischemia–reperfusion via a mechanism that is dependent on eNOS under tight regulation of arginase [44], demonstrating an important role of RBC-derived NO bioactivity in cardiovascular regulation. A cardioprotective effect mediated by RBC eNOS is also supported by the in vivo observation that chimeric mice lacking blood cell eNOS had increased infarct size, resulting in decreased ejection fraction and increased end systolic volume after myocardial ischemia–reperfusion [32]. A recent study using RBC-specific eNOS knockout mice showed that RBC eNOS is cardioprotective in a model of myocardial ischemia–reperfusion [9]. Additional observations suggest that extracellular ATP and purinergic P2 receptor activation may be involved in the activation of eNOS and generation of NO bioactivity in RBCs [6, 12, 40]. A previous study indicated that the P2Y_13_ receptor regulates ATP release from RBCs in human RBCs [42]. Collectively, these observations from experimental studies strongly indicate that RBCs are involved in cardiovascular regulation and that this effect involves export of NO bioactivity and ATP. However, it is unknown whether these beneficial effects of RBCs play a role in human cardiovascular pathology, and it therefore remains to be explored if RBCs play a regulatory role in situations of cardiovascular disease in humans.

ST-segment elevation myocardial infarction (STEMI) occurs when coronary arteries are occluded following plaque rupture and thrombi formation that prevents supply of oxygen and nutrient-rich blood to the myocardium which results in myocardial ischemia with subsequent myocardial dysfunction and necrosis [1]. Although timely reperfusion is essential to salvage the myocardium, it may paradoxically induce further damage, a process referred to as ischemia–reperfusion injury which may account for a significant proportion of the final infarct size [46]. Based on the previous observation that RBCs are able to protect the ischemic myocardium via an eNOS-dependent mechanism [44], it is plausible that RBCs via this mechanism may exert a protective effect to the myocardium in the setting of ischemia–reperfusion in patients with STEMI. Such effect would support a function of RBCs to protect from myocardial injury in acute myocardial infarction.

In this study, we tested the hypothesis that RBCs from STEMI patients protect the heart from ischemia–reperfusion injury through a mechanism that involves the NO–sGC pathway and purinergic signaling. Using well-established ex vivo techniques with RBCs collected from STEMI patients at admission administered to an isolated rat heart model of ischemia–reperfusion, we demonstrate that RBCs from STEMI patients reduce infarct size and improve post-ischemic cardiac function via a mechanism involving purinergic P2Y_13_ receptor-mediated NO–sGC signaling.

## Materials and methods

### Human subjects

Patients with STEMI (chest pain and ST-elevation of > 1 mV in two contiguous leads) and planned for primary percutaneous coronary intervention (PCI) at Karolinska University Hospital were eligible for inclusion. Patients received double antiplatelet therapy with aspirin (300–500 mg) and either ticagrelor (180 mg) or clopidogrel (600 mg) in the ambulance or immediately on arrival to the catheterization laboratory. PCI was performed according to the local clinical routine. Age- and sex-matched healthy subjects without a history of cardiovascular disease were included as controls.

### Animals

Male Wistar rats were purchased from Charles River Laboratories (Wilmington, MA, USA) and housed in the Biomedicum animal facility of Karolinska Institutet until 10–15 weeks of age for experiments before use.

### RBCs’ isolation

Following catheterization of the radial or femoral artery as part of the coronary angiography procedure of the STEMI patients, whole blood was collected in pre-chilled heparin tubes. A first blood sample of 40 ml was collected before starting the coronary intervention, i.e., before reperfusion. An additional sample of 40 ml was collected at the end of the procedure, i.e., after reperfusion. The blood samples were stored at + 4 °C in a refrigerator for up to 6 h before being used in functional experiments (see below). Blood samples from healthy control subjects were collected from an antecubital vein. Following the separation of blood components by centrifugation at 1000*g* and + 4 °C for 10 min, plasma and buffy coat including the top layer of RBCs were discarded to isolate RBCs. After three washing cycles with oxygenated Krebs–Henseleit (KH) buffer (118.5 mM NaCl, 25.0 mM NaHCO_3_, 4.7 mM KCl, 1.2 mM KH_2_PO_4_, 1.2 mM MgSO_4_, 11.1 mM glucose, and 2.4 mM CaCl_2_), purified RBCs were diluted to a hematocrit of ~ 40% with oxygenated (5% CO_2_ in O_2_) KH buffer. This procedure results in the removal of 99% of white blood cells and 98% of platelets [44] and O_2_ saturation of ˃99%. In separate experiments, blood collected from healthy subjects was placed in a refrigerator at + 4 °C for 3 h, 6 h, or 24 h before being washed as above. The RBC-KH buffer suspension was used in the isolated cardiac experiment described below. Samples with hemolysis or clots were excluded.

### Heart isolation and Langendorff perfusion

Wistar rats were anesthetized with pentobarbital sodium (50 mg/kg, i.p.) and heparinization (100 IU/kg, i.v.). The hearts were excised and placed in ice-cold KH buffer. The ascending aorta was then quickly cannulated and perfused with oxygenated KH buffer in a retrograde manner at a constant pressure (100 cm H_2_O) at 37 °C [4]. A balloon-tipped catheter connected to a pressure transducer was inserted into the left ventricle to monitor cardiac functional parameters, including left-ventricular developed pressure (LVDP), and its positive first derivative dP/dt and left-ventricular end-diastolic pressure (LVEDP). During the stabilization period of 30 min, the balloon was given a baseline of LVEDP of 4–10 mmHg, after which baseline parameters were registered. Exclusion criteria were LVDP < 60 mmHg, LVEDP > 10 mmHg, and heart rate < 250 beats/min during the stabilization period in accordance with recent guidelines [4].

### Experimental protocols

The suspension with RBCs from patients with STEMI was incubated with one of the following for 20 min at 37 °C: vehicle [dimethyl sulfoxide, (DMSO) 5 µmol L^−1^; Sigma-Aldrich, St Louis, MO, USA], the soluble guanylyl cyclase inhibitor 1H- [1, 2, 4] oxadiazolo [4,3,-a] quinoxalin-1-one (ODQ, 5 μM; Cayman Chemical, Ann Arbor, MI, USA), the NOS inhibitor N^G^-nitro-L-arginine methyl ester (L-NAME, 100 μM; Sigma-Aldrich, St Louis, MO, USA), the non-selective purinergic P1 receptor antagonist 8-phenyltheophylline (8PT, 10 μM; Sigma-Aldrich, St Louis, MO, USA), the non-selective purinergic P2 receptor antagonist pyridoxal phosphate-6-azo(benzene-2,4-disulfonic acid) tetrasodium salt hydrate (PPADS, 10 μM; Sigma-Aldrich, St Louis, MO, USA), or the P2Y_13_ receptor antagonist MRS2211 (10 μM; Tocris Bioscience, Bristol, UK). In separate experiments, the isolated rat heart was perfused with KH buffer containing vehicle, L-NAME, ODQ, PPADS or the selective inhibitor of cyclic guanosine monophosphate (cGMP)-dependent protein kinase (PKG; KT5823, 1 μM; Cayman Chemical, Ann Arbor, MI, USA) for 10 min before ischemia, after which suspension with RBCs from STEMI patients was given to the heart at the onset of ischemia. Suspension with RBCs from healthy controls was incubated as described above with vehicle, the cell-permeable analog of ATP α-β-methylene ATP (mATP, 100 μM; Cayman Chemical, Ann Arbor, MI, USA) or the combination of mATP and ODQ, PPADS or MRS2211. The oxygenated RBC suspension was then given to the isolated Langendorff-perfused heart at the onset of global myocardial ischemia without an additional washing step (see below). In additional experiments, hearts subjected to 10 min of global ischemia were given KH buffer containing cGMP (100 μM; Sigma-Aldrich, St Louis, MO, USA) at the onset of ischemia after which the heart was collected for immunofluorescence (see below). All experiments including pharmacological interventions were performed with the vehicle as control and using RBCs from the same patient. The concentrations used above were based on pilot studies and previous publications [8, 29, 35, 44].

Global ischemia was induced by clamping the inflow tube, and 3 ml of RBC-KH suspension was administered to the heart via a sidearm connected to the ascending aorta at the onset of ischemia. The duration of global ischemia was 25 min, and during this period, the pre-incubated RBCs were present in the coronary circulation. Reperfusion, which rinsed away the RBCs, was initiated by releasing the clamp and was maintained for 60 min. Previous detailed investigations have revealed that human RBCs can be administered to the isolated rat heart without affecting cardiac functional recovery per se [44, 45].

### Determination of heart infarct size

At the end of reperfusion, hearts were frozen at − 20 °C and sectioned into 1 mm-thick slices from the apex to the base, stained with triphenyltetrazolium chloride for 15 min, and fixed in 4% formaldehyde for 18 h. Necrotic negatively stained myocardium was measured using Adobe Photoshop Elements 2019 Edition by an investigator blinded to group allocation [4].

### Immunofluorescence

For the detection of P2Y_13_ receptor, isolated RBCs (1%) were incubated on glass coverslips overnight at 37ºC in Roswell Park Memorial Institute Medium (RPMI) as described elsewhere [31]. RBCs were fixed with 4% paraformaldehyde and blocked in a phosphate-buffered saline (PBS) solution containing 3% bovine serum albumin (BSA). Then, RBCs were incubated at + 4 ℃ overnight with the following primary antibodies in a 3% BSA/PBS solution: a rabbit monoclonal anti-P2Y_13_ (1:100 dilution, catalog No. ab108444; Abcam, Cambridge, UK), a rabbit polyclonal anti-P2Y_13_ (1:100 dilution, catalog No. PA5-77,675; Thermo Fisher Scientific, Waltham, MA, USA), or with a proper matched control (rabbit IgG; Abcam, Cambridge, UK), followed by incubation with the secondary antibody Alexa-Fluor™ 488-conjugated goat anti-rabbit (1:750 dilution; Life Technologies, Carlsbad, CA, USA) at room temperature for 1 h.

For the detection of PKG-dependent phosphorylation of vasodilator-stimulated phosphoprotein (pVASP), Langendorff-perfused rat hearts were collected after 30 min of stabilization and 10 min of ischemia followed by 1 min of reperfusion. The hearts were then perfused with 4% formaldehyde and fixed for 24 h at room temperature, dehydrated in graded ethanol (70, 95, and 99%), embedded in paraffin, sectioned using a microtome, and mounted on coated glass slides (Superfrost^®^ plus; Thermo Fisher Scientific, Waltham, MA, USA). At least 12 slides containing ~ 4 tissue cross-Sects. (5 μm thick) from each animal were examined. Sections were deparaffinized in xylene and rehydrated in graded ethanol. For antigen retrieval, slides were subjected to high-pressure boiling in citrate buffer (pH 6.0). Heart cross-sections were permeabilized with 0.3% Triton X-100 for 10 min, blockade with goat serum (Abcam, Cambridge, UK), and incubated overnight (+ 4 °C) with the following primary antibodies: a rabbit polyclonal anti-phosphorylated vasodilator-stimulated phosphoprotein (pVASP) (Ser239; 1:100 dilution, IgG, catalog No. PA5-99,377; Thermo Fisher Scientific, Waltham, MA, US) and a mouse monoclonal anti-sarcomeric alpha-actinin antibody (1:100 dilution, IgG1, catalog No. ab9465; Abcam, Cambridge, UK). Specific labeling was detected with an Alexa-Fluor™ 488 goat anti-rabbit (1:200 dilution, Life Technologies, Carlsbad, CA, USA) or an Alexa-Fluor™ 594 goat anti-mouse (1:200 dilution, Life Technologies, Carlsbad, CA, USA), respectively. Cell nuclei were counterstained with Hoechst dye (Sigma-Aldrich, St Louis, MO, USA). To confirm the specificity of antibodies, isotype controls were used as negative controls (rabbit IgG or mouse IgG, both from Abcam, Cambridge, UK). Fields were captured with the fluorescence microscope equipped with an × 40 objective lens and an × 10 eyepiece (Leica DM3000 digital microscope; Leica Biosystems, Wetzlar, Germany), digitized, and analyzed (ImageJ software 1.53v, Bethesda, MA, USA).

### Statistical analysis

LVDP and dP/dt are presented as the percentage of recovery during reperfusion from baseline levels. LVEDP is expressed in absolute value. Normal distribution of data was analyzed using D’Agostino and Pearson normality test. Differences between two groups were analyzed by paired or unpaired Student’s *t* test and Mann–Whitney test depending on the matching and distribution. Cardiac performance during the reperfusion period was analyzed by stacked 2-way ANOVA with repeated measures for both treatment and time for dependent observations or ordinary 2-way ANOVA for independent observations followed by Tukey’s or Dunnett’s post hoc test. All statistical analyses were calculated using GraphPad Prism version 7.04 (GraphPad, San Diego, CA, USA). Data are unless otherwise stated presented as mean ± standard deviation (SD). Infarct size is presented as mean ± SD and individual data points. *P* < 0.05 was considered as statistically significant.

## Results

### Study subject characteristics

Study subject characteristics are shown in Table 1. Patients with STEMI had a significantly higher body mass index, whereas they had lower total cholesterol, high-density lipoprotein cholesterol, and low-density lipoprotein cholesterol levels in comparison with healthy subjects. None of the healthy subjects took any medications or had any history of cardiovascular disease.

**Table 1:** Clinical characteristics of study subjects

	Healthy subjects (*n* = 23)	STEMI (*n* = 48)
Age, years	63 ± 7	69 ± 12*^b^
Male, *n* (%)	14 (61)	38 (79)
Smokers, *n* (%)	1 (4)	15 (31)**^c^
BMI, kg/m^2^	23 ± 2.9	27 ± 4.1***^a^
SBP, mmHg	128 ± 16	130 ± 22
DBP, mmHg	82 ± 7	79 ± 12
Hemoglobin, g/l	140 ± 11	135 ± 15
Erythrocyte count, *10^12^ cells/l	4.6 ± 0.4	4.3 ± 0.5
EVF, %	41 ± 3	39 ± 4*^a^
MCV, fl	90 ± 2	90 ± 5
MCH, pg	30 ± 1	30 ± 2
Leukocyte count, × 10^9^ cells/l	4.7 ± 0.6	11.1 ± 4.8***^b^
Platelet count, × 10^9^ cells/l	222 ± 40	238 ± 57
Creatinine, µmol/l	79 ± 15	87 ± 47
Triglycerides, mmol/l	1.3 ± 0.5	1.7 ± 1.2
Total cholesterol, mmol/l	5.5 ± 0.8	4.8 ± 1.3**^a^
HDL-c, mmol/l	1.6 ± 0.4	1.1 ± 0.3***^a^
LDL-c, mmol/l	3.3 ± 0.6	2.9 ± 1.1*^a^
Troponin T, ng/l	NA	4981 ± 5962
Comorbidities, *n*		
Hypertension	0	21
Dyslipidemia	0	6
Diabetes (HbA1c, mmol/mol)	0 (NA)	8 (54 ± 8.7)
Medications at inclusion, *n*		
Aspirin	0	48
Ticagrelor	0	37
Clopidogrel	0	9
Cangrelor	0	2
ACEi/ARB	0	19
Lipid lowering	0	11
β-blockers	0	9
Calcium channel i	0	11
Culprit vessel		
LAD	NA	25
LCX	NA	6
RCA	NA	17
Symptom to balloon time, min	NA	455 ± 360
Contrast dye, ml	NA	148 ± 65
Ejection fraction, %	NA	47 ± 9

Values are mean ± SD. *ACEi* angiotensin-converting enzyme inhibitor, *ARB* angiotensin receptor blocker, *BMI* body mass index, *DBP* diastolic blood pressure, *EVF* erythrocyte volume fraction, *HbA1c* hemoglobin A 1c, *HDL-c* high-density lipoprotein cholesterol, *LAD* left anterior descending artery, *LCX* left circumflex artery, *LDL-c* low-density lipoprotein cholesterol, *MCH* mean corpuscular hemoglobin, *MCV* mean corpuscular volume, *NA* not applicable, *RCA* right coronary artery, *SBP* systolic blood pressure, *STEMI* ST-elevation myocardial infarction

*P < 0.05, **P < 0.01, and ***P < 0.001

^a^Unpaired Student’s *t* test

^b^Mann–Whitney test

^c^Fisher’s exact test

### RBCs from STEMI patients protect against cardiac ischemia–reperfusion injury

Baseline pre-ischemic cardiac function and heart rate of all the isolated rat hearts are shown in Supplementary Table 1.

**Fig. 1:**
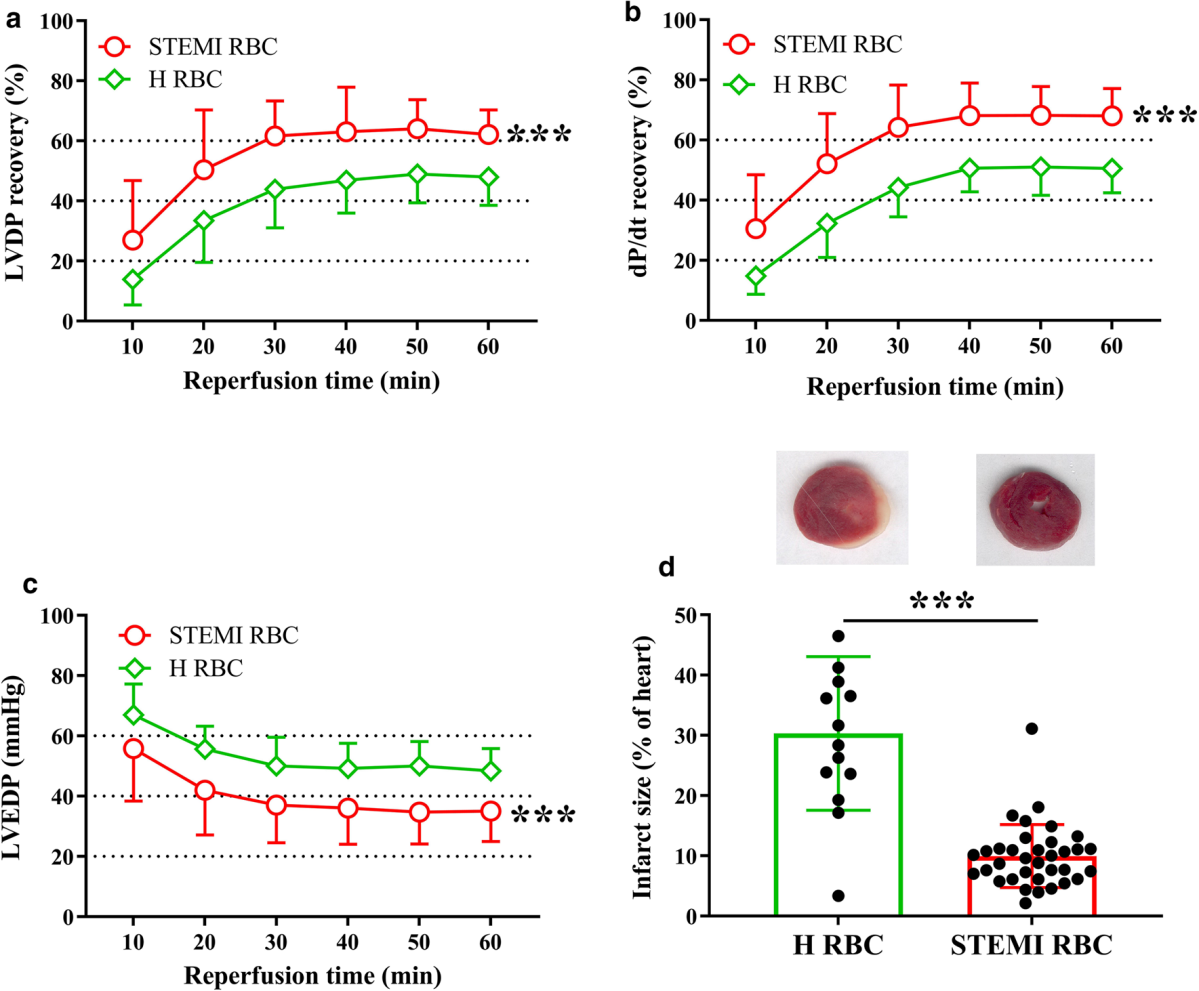
Red blood cells (RBCs) from patients with ST-elevation myocardial infarction (STEMI) protect the heart from ischemia–reperfusion injury. Effect of RBCs on recovery of cardiac function and infarct size in isolated rat hearts subjected to global ischemia–reperfusion. Recovery of **a** left-ventricular developed pressure (LVDP), **b** positive dP/dt, **c** left-ventricular end-diastolic pressure (LVEDP), and **d** myocardial infarct size of heart following administration of RBCs from patients with STEMI (STEMI RBC) (*n* = 35) and healthy subjects (H RBC) (*n* = 23). Post-ischemic LVDP and dP/dt are presented as percentage recovery from baseline and LVEDP in absolute pressure. Data are presented as mean ± SD. Statistical differences in **a**–**c** were analyzed with 2-way ANOVA including all time points. Mann–Whitney test was performed in (**d**). ***P < 0.001 vs. H RBC

First, we investigated the functional role of RBCs from patients with STEMI on cardiac post-ischemic functional recovery and infarct size. Administration of RBCs from patients with STEMI in Langendorff-perfused rat hearts significantly improved post-ischemic recovery of LVDP (Fig. 1a) and dP/dt (Fig. 1b), and reduced LVEDP (Fig. 1c) in comparison with RBCs from healthy controls. In addition, myocardial infarct size was significantly smaller in hearts given RBCs from STEMI patients (Fig. 1d). There was no difference in the recovery of LVDP irrespective of whether the RBCs were collected before or after reperfusion (Supplementary Fig. 1a). The presence of hypertension (Supplementary Fig. 1b), type 2 diabetes (Supplementary Fig. 1c), or dyslipidemia (Supplementary Fig. 1d) did not significantly alter the effect of RBCs on post-ischemic recovery of LVDP. The post-ischemic recovery was comparable irrespective of whether RBCs from STEMI patients pretreated with ticagrelor or clopidogrel were administered to the heart (Supplementary Fig. 2a). Baseline medication including angiotensin-converting enzyme inhibitor, angiotensin receptor antagonist, or calcium channel inhibitor did not affect the protective effect of RBCs from STEMI patients (Supplemental Fig. 2b, c). Moreover, storage of RBCs from healthy subjects for 3–24 h at + 4 °C in a refrigerator did not affect post-ischemic recovery of LVDP (Supplementary Fig. 3). This indicates that the duration between RBCs collection and performance of functional experiment did not affect the recovery of cardiac function. Collectively, these results suggest that RBCs from patients with STEMI protect from cardiac ischemia–reperfusion injury.

### The cardioprotection induced by RBCs from STEMI patients is mediated by the NO–sGC pathway

**Fig. 2:**
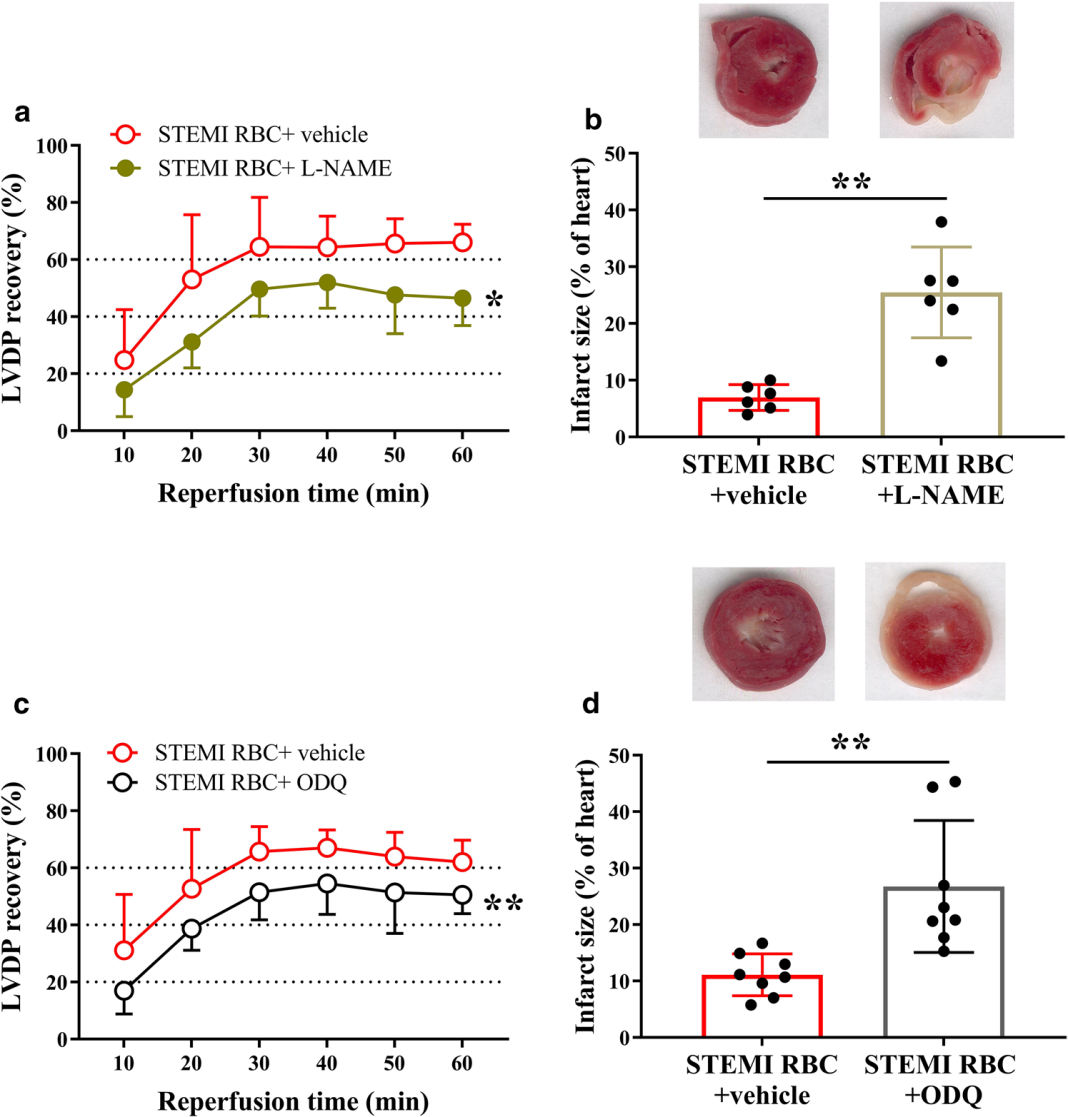
RBCs from patients with STEMI protect from ischemia–reperfusion injury via nitric oxide synthase (NOS) and soluble guanylyl cyclase (sGC) signaling. Post-ischemic recovery of LVDP and infarct size in hearts following administration of RBCs from STEMI patients incubated with (**a**, **b**) vehicle (*n* = 6) or the NOS inhibitor L-NAME (*n* = 6); (**c**, **d**) vehicle (*n* = 8) or the sGC inhibitor ODQ (*n* = 8). Post-ischemic LVDP is presented as percentage recovery from baseline and infarct size as a percentage of the heart. Data are presented as mean ± SD. Statistical differences were analyzed with 2-way ANOVA including all time points in (**a**, **c**) and Student’s *t* test in (**b**, **d**). *P < 0.05, **P < 0.01 vs. vehicle

**Fig. 3:**
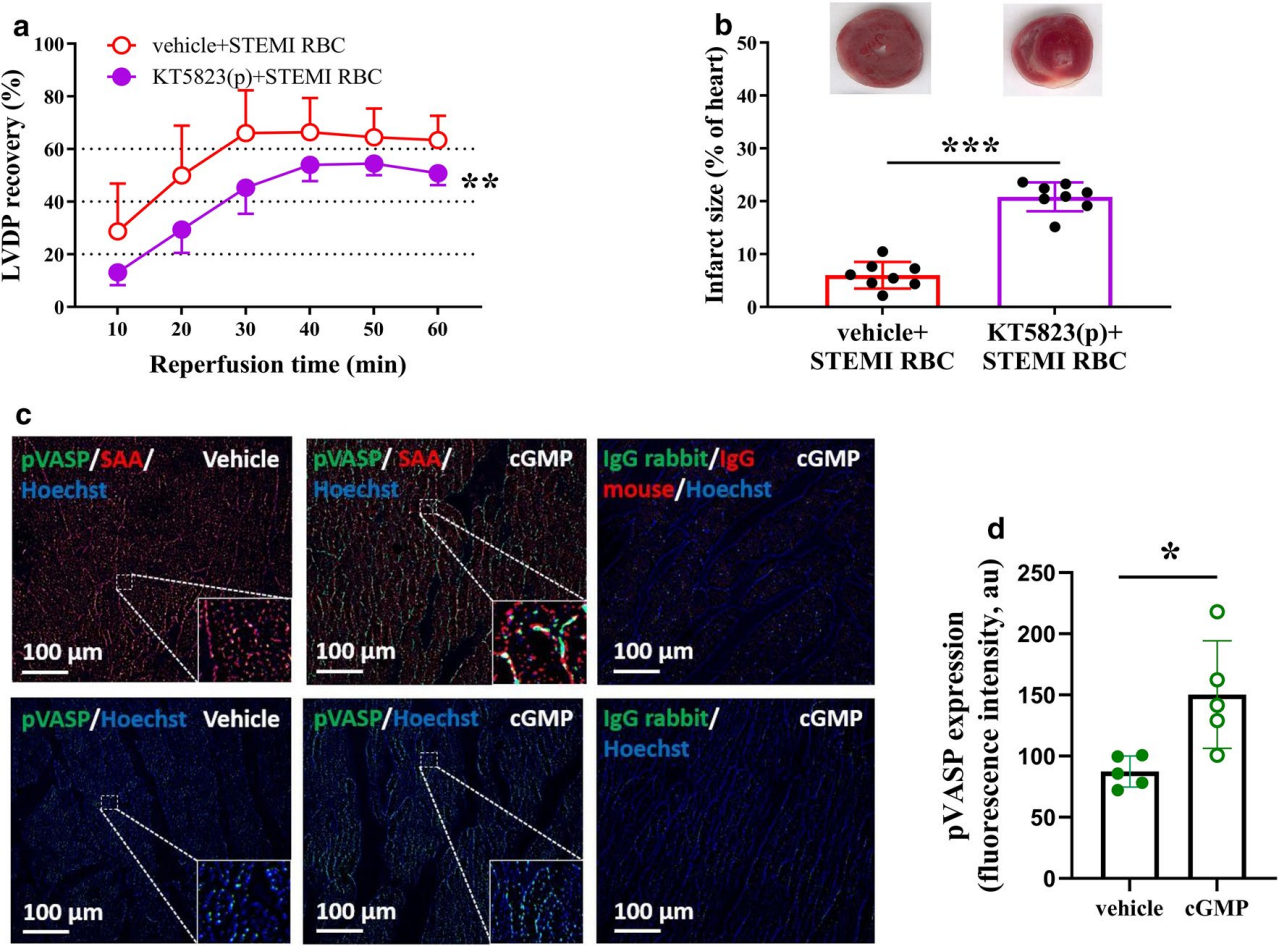
RBCs from patients with STEMI protect from ischemia–reperfusion injury via cardiac protein kinase G (PKG). Post-ischemic recovery of LVDP **a** and infarct size **b** in heart perfused (p) with vehicle (*n *= 8) or the PKG inhibitor KT5823 (*n* = 8) in Krebs–Henseleit (KH) buffer prior to administration of RBCs from STEMI patients. Representative immunofluorescence images **c** and quantification in an arbitrary units (au) **d** showing colocalization of phosphorylated vasodilator-stimulated phosphoprotein (pVASP)/sarcomeric alpha-actinin (SAA) expression in the heart tissue. Immunoreactivity was visualized using Alexa Fluor™ 594 secondary antibody (SAA, red) and Alexa Fluor™ 488 antibody (pVASP, green). Nuclei were stained with Hoechst (blue). Statistical differences were analyzed with 2-way ANOVA including all time points in (**a)** and Student’s *t* test in (**b**, **d**). *P < 0.05, **P < 0.01, ***P < 0.001 vs. vehicle

Next, we investigated the mechanisms by which RBCs from STEMI patients improve cardiac post-ischemic recovery. To test whether the protective effect involves the NO–sGC pathway, RBCs from STEMI patients were pre-incubated with the NOS inhibitor L-NAME before being given to the isolated heart. L-NAME significantly attenuated the improvement in post-ischemic recovery induced by RBCs from STEMI patients and increased infarct size (Fig. 2a, b). In addition, pre-incubation of the RBCs from patients with STEMI with the sGC inhibitor ODQ attenuated the recovery of LVDP and increased infarct size (Fig. 2c, d). To determine the signaling in the myocardium, the hearts were perfused with the PKG inhibitor KT5823 before the administration of the RBCs from STEMI patients. This led to attenuation of the recovery of LVDP and increased infarct size (Fig. 3a, b). By contrast, perfusion of the isolated heart with L-NAME or ODQ before the administration of RBCs from patients with STEMI did not affect cardiac post-ischemic recovery (Supplementary Fig. 4). Pre-incubation of RBCs from healthy controls with L-NAME and ODQ did not affect the post-ischemic LVDP recovery (Supplementary Fig. 5a, b). Pre-incubation of RBCs from healthy controls with KT5823 or perfusion of the hearts with KT5823 before administration of RBCs from healthy controls did not affect LVDP recovery (Supplementary Fig. 5c, d). To determine whether cGMP present in the coronary circulation is able to activate cardiac PKG, the expression of pVASP at Ser239, which is the major phosphorylation site of PKG, was detected in cardiac tissue. Administration of cGMP to the isolated hearts significantly increased cardiac expression of pVASP, with expression localized in cardiomyocytes (Fig. 3c, d). These data suggest that the cardioprotective effect of RBCs from patients with STEMI is mediated by NOS signaling via activation of sGC in the RBCs and cardiac PKG.

### Cardioprotection induced by RBCs from STEMI patients is regulated through activation of P2Y_13_ signaling

**Fig. 4:**
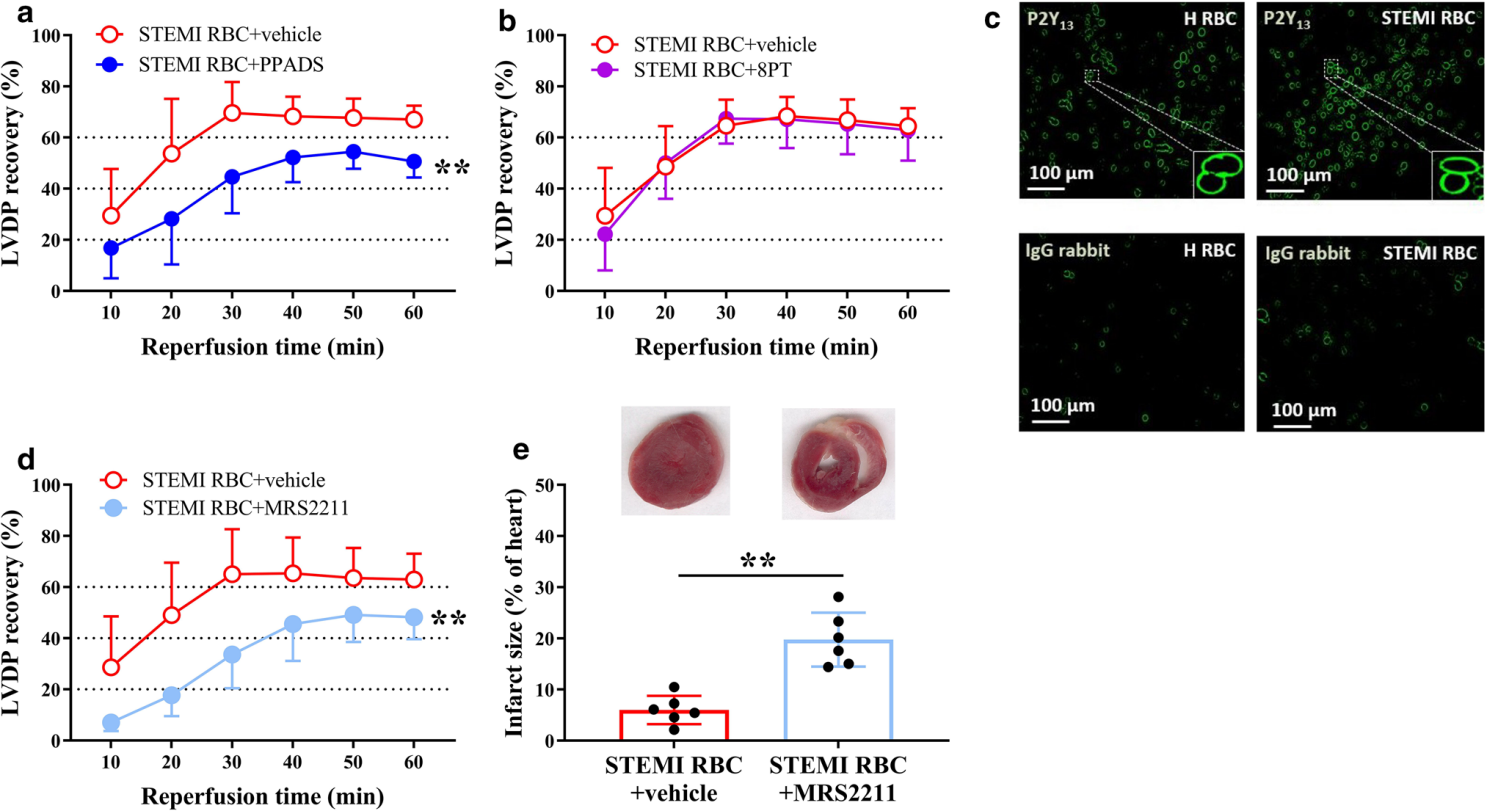
RBCs from patients with STEMI protect from ischemia–reperfusion injury via the purinergic P2Y_13_ receptor. Post-ischemic recovery of LVDP **a**, **b**, **d** and infarct size **e** in isolated rat heart subjected to global ischemia–reperfusion following administration of RBCs from STEMI patients (STEMI RBC). The RBCs were incubated with **a** vehicle (*n* = 7) or the purinergic P2 receptor antagonist PPADS (*n* = 7); **b** vehicle (*n* = 11) or the purinergic P1 receptor antagonist 8PT (*n* = 11); **d** vehicle (*n* = 6) or the P2Y_13_ receptor antagonist MRS2211 (*n* = 6) before being administered to the heart. **c** Representative immunofluorescence images showing P2Y_13_ receptor expression in RBCs from patients with STEMI (STEMI RBC) and healthy subjects (H RBC). Immunoreactivity was visualized using Alexa Fluor™ 488 antibody (P2Y_13_, green). Post-ischemic LVDP is presented as a percentage recovery from baseline and infarct size as a percentage of the heart. Data are presented as mean ± SD. Statistical differences were analyzed with 2-way ANOVA including all time points in a, b and d or Student’s *t* test in (**e**). **P < 0.01 vs. vehicle

To investigate the involvement of ATP and purinergic receptors in the activation of the NO–sGC pathway in RBCs from STEMI patients, the RBCs were pre-incubated with the non-selective P1 receptor antagonist 8PT and the P2 receptor antagonist PPADS. The cardioprotective effect was abolished in hearts given RBCs pre-incubated with PPADS (Fig. 4a). In contrast, administration of RBCs pre-incubated with 8PT failed to alter the post-ischemic recovery (Fig. 4b). Based on the previous finding that mRNA for the P2Y_13_ receptor is expressed in human RBCs [42], we detected the protein expression of the receptor. We found that the P2Y_13_ receptor is abundantly expressed in RBCs from STEMI patients and healthy controls (Fig. 4c, Supplementary Fig. 6a, b). Pre-incubation of RBCs with the P2Y_13_ receptor antagonist MRS2211 resulted in attenuation of the cardiac post-ischemic recovery and increased infarct size compared to vehicle (Fig. 4d, e). To distinguish whether PPADS blocked P2 receptors in RBCs or the coronary circulation, cardiac P2 receptors were blocked by perfusing with PPADS in KH buffer for 10 min followed by administration of RBCs at the onset of ischemia. Post-ischemic recovery of LVDP was not affected by perfusion with PPADS in comparison with vehicle (Supplementary Fig. 7a). Pre-incubation of PPADS or MRS2211 with RBCs from healthy controls did not affect the cardiac post-ischemic LVDP recovery (Supplementary Fig. 8a, b). Collectively, these results indicate that the beneficial effect is regulated by P2Y_13_ receptor activation in RBCs from STEMI patients.

### Extracellular ATP-activated P2 signaling and NO–sGC pathway under ischemic condition

**Fig. 5:**
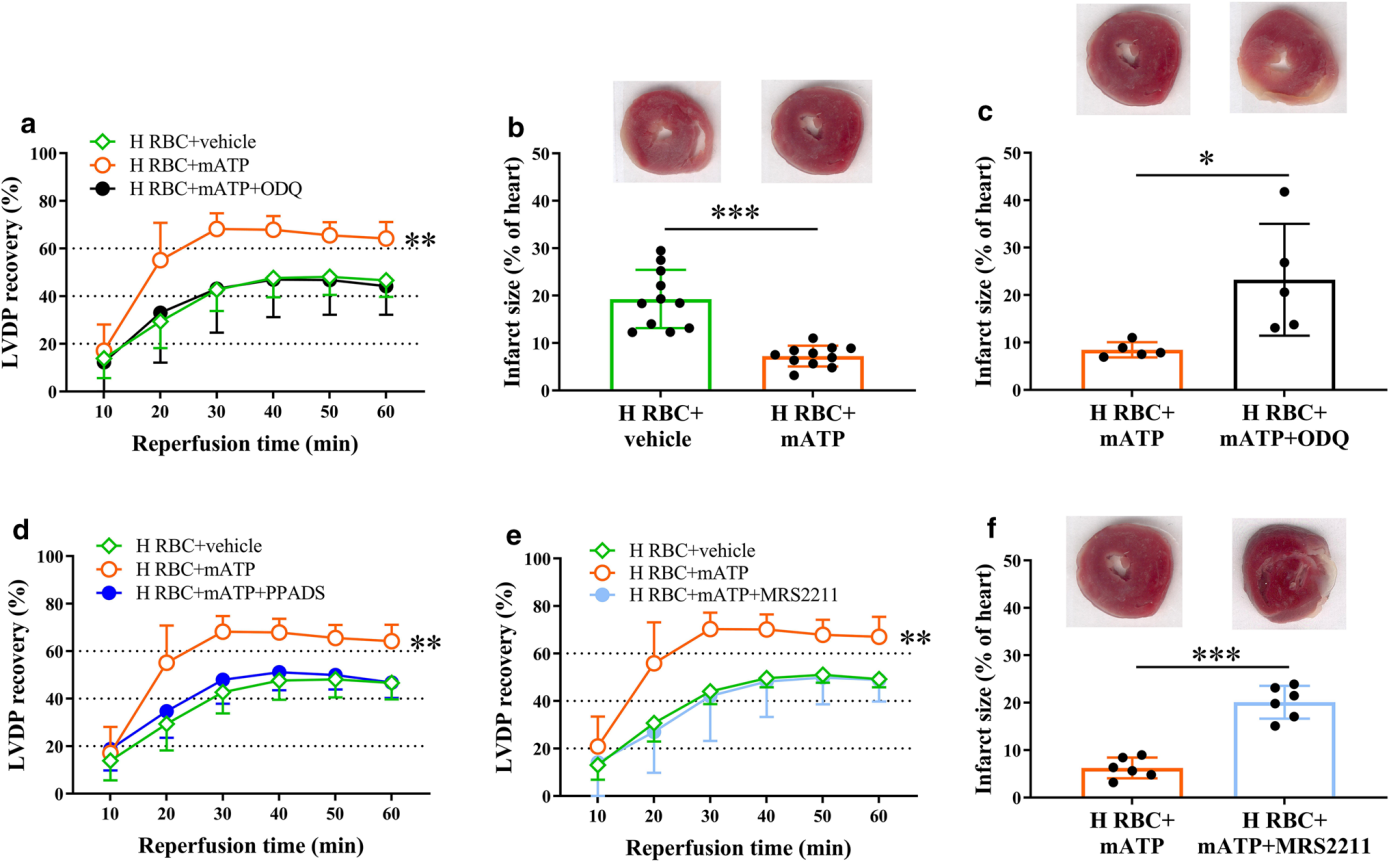
ATP induces protective signaling in RBCs via the P2Y_13_ receptor and sGC. Recovery of LVDP (**a**, **d**, **e**) and infarct size (**b**, **c**, **f**) following administration of RBCs from healthy subjects (H RBC) incubated with vehicle (*n* = 6), the stable and cell-permeable ATP analog mATP (*n* = 6), mATP + ODQ (*n* = 5), mATP + PPADS (*n* = 5), or mATP + MRS2211 (*n* = 6). Post-ischemic LVDP is presented as percentage recovery from baseline and infarct size as a percentage of the heart. Data are presented as mean ± SD. Statistical differences were analyzed with 2-way ANOVA including all time points in (**a**, **d**, **e)** or Student’s *t* test in (**b**, **c**, **f**). *P < 0.05, **P < 0.01, ***P < 0.001 vs. vehicle or mATP

To determine whether extracellular ATP protects the heart and activates the NO–sGC signaling pathway in RBCs via the P2 receptor, RBCs from healthy subjects were pre-incubated with mATP, a cell-permeable and stable ATP analog, to active P2 signaling. Post-ischemic recovery of LVDP was significantly improved and infarct size was reduced in hearts given RBCs incubated with mATP compared to vehicle-incubated RBCs (Fig. 5a, b). This beneficial effect of mATP was abolished by pre-incubation with ODQ, PPADS, and MRS2211 (Fig. 5a–f).

## Discussion

**Fig. 6:**
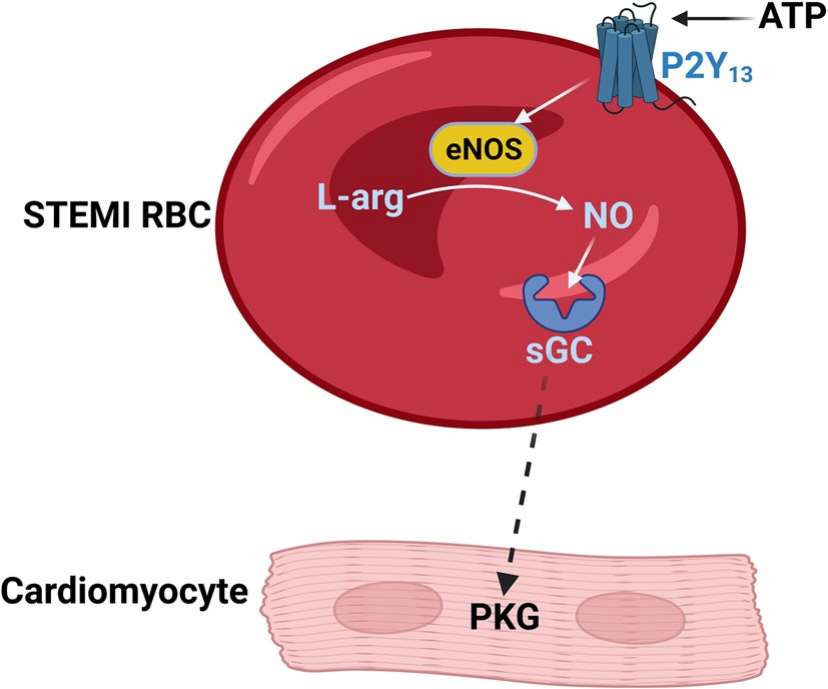
Schematic illustration of the proposed signaling in RBCs from patients with STEMI. RBCs from patients with STEMI induce cardioprotective effects via a mechanism dependent on NOS activation and sGC. Activation of purinergic signaling by ATP via the P2Y_13_ receptor results in activation of the NO–sGC pathway within the RBCs which leads to the signaling of still unexplored mechanism(s) (dashed arrow) to activate PKG in the cardiomyocytes resulting in cardioprotection. *ATP* adenosine triphosphate, *eNOS* endothelial nitric oxide synthase, *L-arg* L-arginine, *NO* nitric oxide, *PKG* protein kinase G, *RBCs* red blood cells, *sGC* soluble guanylyl cyclase, *STEMI* ST-elevation myocardial infarction

Previous studies have indicated that RBCs are involved in physiological cardiovascular regulation by exporting vasodilator NO bioactivity [11, 34, 39]. Results from experimental studies also suggest that the export of NO bioactivity from RBCs exerts cardioprotective effects via an eNOS-dependent mechanism [44]. However, it has remained unexplored whether this effect of RBCs provides protection against myocardial injury in the setting of STEMI. The current results demonstrate that RBCs from STEMI patients markedly improved cardiac post-ischemic recovery and reduced myocardial infarct size in an ex vivo isolated heart model of ischemia–reperfusion. The cardioprotective effect was abolished by inhibition of the NO–sGC signaling pathway in RBCs and cardiac PKG, as well as by P2Y_13_ receptor blockade in the RBCs. In addition, purinergic receptor stimulation in RBCs from healthy subjects protected from ischemia–reperfusion injury via an action mediated by the P2Y_13_ receptor and an sGC-dependent mechanism. These results demonstrate a novel function of RBCs from STEMI patients leading to cardioprotection with improved cardiac tolerance to ischemia–reperfusion and reduced infarct size in an isolated rat heart model. This effect is mediated via a mechanism that involves P2Y_13_ receptor stimulation which subsequently activates the NO–sGC pathway in RBCs and cardiac PKG (Fig. 6).

RBCs are not only important for the transport of respiratory gases, but are also critically involved in cardiovascular homeostasis as a regulator of cardiovascular function by the export of ATP and NO bioactivity [16, 38]. Emerging evidence suggest that RBCs express a functional eNOS and downstream signaling pathways involved in NO signaling, including sGC [8, 23]. It should also be noted that NO can be formed via NOS-independent pathways, a mechanism that may be particularly relevant during ischemia [26, 30]. The concept that RBCs generate biologically active NO has been controversial based on the opinion that NO bioactivity is rapidly scavenged by oxy- and deoxyhemoglobin within the RBC. However, the importance of eNOS in RBCs for vascular regulation was recently confirmed by the finding that mice lacking eNOS specifically in RBCs had elevated blood pressure, and rescue of eNOS in RBCs of global eNOS knockout mice resulted in reduction of blood pressure [25]. Furthermore, the critical cardioprotective role of eNOS in RBCs during ischemia–reperfusion has been shown in isolated hearts given RBCs from eNOS knockout mice [44] and in vivo using RBC-specific eNOS knockout mice [9]. Based on these observations, we hypothesized that the cardioprotection induced by RBCs from STEMI patients involves eNOS and downstream sGC activation. Accordingly, we found that the post-ischemic recovery of cardiac function was attenuated following inhibition of eNOS as well as following inhibition of sGC. By contrast, perfusion of the isolated heart with the sGC inhibitor ODQ before administration of the RBCs failed to induce protection to the heart. These results clearly indicate that the cardioprotective effect of the RBCs from STEMI patients was mediated via increased signaling of the NO–sGC pathway within the RBCs. The NO–sGC signaling pathway is known to induce cardioprotection via activation of cardiac PKG [20]. Accordingly, the cardioprotective effect of RBCs from STEMI patients was attenuated by inhibition of PKG in the heart, suggesting that cardiac PKG stimulated by the activated NO–sGC signaling in RBCs from STEMI patients plays a key role in the cardioprotection.

The molecular identity of the mediator that is released from RBCs and mediates the cardioprotection is unclear. Since oxygenated hemoglobin reacts very rapidly with NO to form methemoglobin and nitrate, it is considered unlikely that NO itself can be released from RBCs. According to other theories, NO is released in the form of nitrate [10] or as an S-nitrosothiol [22]. However, these theories do not fit with the findings in the current study, suggesting that the protective effect is dependent on sGC activation in the RBC. Thus, it is likely another still unidentified signaling molecule that mediates the NO bioactivity exported from RBCs. Interestingly, it is known that cGMP can be exported from several cell types including RBCs [14]. From our current study, we also observed that exogenous cGMP, which is the downstream molecule of NO–sGC signaling, stimulates the PKG activation in cardiomyocytes. However, it remains unclear whether release of cGMP from RBCs occurs in response to stimulation of sGC and can mediate the cardioprotective effect of RBCs in the present study.

Extracellular ATP has been shown to active eNOS and increases intracellular NO production in RBCs [40]. Furthermore, the release of ATP has been shown to prevent reperfusion injury [18]. We therefore investigated whether ATP may be involved in the cardioprotective effect of RBCs from STEMI patients. Purinergic signaling involves the activation of membrane-bound P1 and P2 receptors by extracellular nucleosides and nucleotides including adenosine and ATP, respectively [37, 47]. Previous observations suggest that P2 receptors including cardiomyocyte P2Y_2_ and P2Y_11_ receptors mediate cardioprotection [2, 21]. However, less is known regarding the role of purinergic receptors on RBCs. Our results show that incubation of the RBCs with a P2 receptor antagonist, but not a P1 receptor antagonist, abolished the cardioprotection, which is supported by the previous demonstration of P2 receptors expressed on RBCs [5]. In the current study, we show that P2Y_13_ receptor is abundantly expressed in RBCs from STEMI patients and healthy controls. Moreover, pre-incubation with a P2Y_13_ receptor antagonist with the RBCs also blocked the protective effect, indicating that the cardioprotective effect involves activation of the P2Y_13_ receptor. We further specified that the cardioprotective effect derived from activation of P2 signaling in RBCs, but not in the heart, by perfusing isolated heart with P2 antagonist in buffer prior to administration of RBCs at the onset of ischemia. Although the data suggest that the P2 receptors are activated by extracellular ATP, it remains to be established which type of cell ATP originates from and how ATP is transmitted to target RBCs to elicit the cardioprotection. The RBC is a potential source, since previous studies have demonstrated that ATP is released from RBCs under hypoxic conditions [17] as well as from endothelial cells in response to blood flow changes and during hypoxia, which are conditions characteristic of STEMI patients [3]. We demonstrate that incubation of healthy RBCs with extracellular ATP mimicked the cardioprotective effect of RBCs from STEMI patients, further supporting a protective action of ATP. It is also known that export of NO bioactivity, in addition to ATP, from RBC is stimulated under hypoxic conditions [11]. Importantly, the cardioprotective effect of extracellular ATP was blocked by the sGC inhibitor ODQ and the P2Y_13_ receptor antagonist MRS2211 supporting a link between purinergic signaling and the NO–sGC pathway in RBCs (Fig. 6).

The present results may also have potential implications for endogenous protection from ischemia–reperfusion injury in STEMI. Experimental studies have repeatedly shown that remote ischemic conditioning results in cardioprotection with reduced infarct size as endpoint [13, 19]. However, the mechanisms triggering and mediating the cardioprotective effect remain unresolved, which may explain why the robust cardioprotection in experimental animal models to the clinical setting has been challenging [19, 24]. The present results demonstrate that RBCs from patients with STEMI protect against ischemia–reperfusion injury in a remote cardiac preparation may indicate that RBCs are able to transfer a cardioprotective signal. Whether RBCs may be involved in remote ischemic conditioning needs to be further investigated in future studies.

Cardiovascular risk factors, such as type 2 diabetes [33, 45], anemia [43], and chronic kidney disease [15], have been observed to be associated with impaired RBC function and NO bioactivity. This RBC dysfunction observed among patients with type 2 diabetes has been shown to result in impairment of cardiac function and larger infarct size [45]. It may therefore appear surprising that we observed a protective effect of RBCs from patients with STEMI in the present study with no apparent difference in post-ischemic cardiac recovery between STEMI patients with and without type 2 diabetes. Although this study was not powered for analysis of subgroups of co-morbidities, this may be due to the observation that the patients with type 2 diabetes in the present study had comparably well-controlled diabetes with a mean HbA1c of 54 mmol/mol. This would be in line with previous studies, demonstrating that the negative effect of RBCs in type 2 diabetes is attenuated among type 2 diabetes patients with improved glycemic control [28, 33].

The present study has certain limitations that deserve consideration. First, the study was performed on isolated rat hearts in an ex vivo model and any extrapolation to how RBCs act in STEMI patients in vivo should be made with caution. A clear advantage of the ex vivo model is that the function of the RBCs collected from patients can be studied and investigated in detail. Second, most of the data describing the signaling pathways were based on experiments involving pharmacological tools. Experiments using genetic deletion or gene silencing are unfortunately not possible when using human red blood cells. Third, it cannot be excluded that medication given to the STEMI patients affects RBCs. P2Y_12_ receptor antagonists, including ticagrelor which has been suggested to exert cardioprotective effects by inhibition of adenosine reuptake in RBCs [41], are according to clinical routine administered to STEMI patients in the ambulance. However, we observed no difference in the protective effects of RBCs between patients treated with ticagrelor or clopidogrel, suggesting that ticagrelor did not contribute to the protective effect. This is in line with the finding the P1 receptor antagonist 8PT did not affect the cardioprotective effect of the RBCs from patients with STEMI. Furthermore, additional baseline medication did not appear to have an impact on the protective effect of the RBCs, which is in line with a previous study, indicating that co-medication in patients with type 2 diabetes did not seem to influence RBC function [48, 49]. It should be emphasized, however, that the analyses of subgroups depending on co-morbidity and co-medication should be interpreted with caution due to the limited number of patients. Finally, although our results show that RBCs from healthy subjects could be stored for up to 24 h without affecting cardiac post-ischemic recovery, it cannot be excluded that the storage (≤ 6 h) of RBCs from STEMI patients influenced the function of those RBCs.

## Conclusion

Our study demonstrates a previously unknown cardioprotective effect mediated by RBCs from patients with STEMI. As illustrated in Fig. 6, the cardioprotection is mediated via activation of purinergic signaling via the P2Y_13_ receptor and the NO–sGC pathway within the RBCs and PKG in the heart. These novel findings improve our understanding of the significant role of RBCs in cardiovascular regulation and as an endogenous protective mechanism in ischemic heart disease.

## Supplementary Information

Below is the link to the electronic supplementary material.

Supplementary file1 Fig. 1 Effect of reperfusion intervention or major comorbidities of patients with ST-elevation myocardial infarction (STEMI) on recovery of left ventricular developed pressure (LVDP) in isolated rat hearts subjected to global ischemia-reperfusion. Recovery of LVDP in heart following administration of red blood cells (RBCs) from STEMI patients (STEMI RBC) (a) before (n=35) or after (n=25) reperfusion; (b) with (n=16), or without (n=19) hypertension; (c) with (n=8), or without (n=27) type 2 diabetes (T2D); (d) with (n=5), or without (n=30) dyslipidemia compared to that in heart given RBCs from healthy controls (H RBC) (n=23). Post-ischemic LVDP is presented as percentage recovery from baseline. Data are presented as mean ± SD. Statistical differences were analyzed with 2-way ANOVA including all time points (JPG 2694 KB)

Supplementary file2 Fig. 2 Effect of baseline medication on recovery of LVDP in isolated rat hearts subjected to global ischemia-reperfusion. Recovery of LVDP following administration of RBCs from STEMI patients (STEMI RBC) treated (a) with ticagrelor (n=28) and clopidogrel (n=7); (b) with (n=14) or without (n=21) angiotensin converting enzyme inhibitor or angiotensin receptor blocker (ACEi/ARB); (c) with (n=7) or without (n=28) calcium channel inhibitor (calcium channel i). Post-ischemic LVDP is presented as percentage recovery from baseline. Data are presented as mean ± SD. Statistical differences were analyzed with 2-way ANOVA including all time points (JPG 2357 KB)

Supplementary file3 Fig. 3 Effect of duration between RBCs collection and experimental determination of recovery of LVDP in isolated hearts subjected to global ischemia-reperfusion. Recovery of LVDP following administration of RBCs from healthy subjects (H RBC) after placing for 3 h (n=7), 6 h (n=8) or 24 h (n=7) at +4°C. Post-ischemic LVDP is presented as percentage recovery from baseline. Data are presented as mean ± SD. Statistical differences were analyzed with 2-way ANOVA including all time points (JPG 655 KB)

Supplementary file4 Fig. 4 Post-ischemic recovery of LVDP in hearts perfused (p) with vehicle (n=7), L-NAME (n=6) or ODQ (n=6) in KH buffer prior to administration of RBCs from STEMI patients. Post-ischemic LVDP is presented as percentage recovery from baseline. Data are presented as mean ± SD (JPG 716 KB)

Supplementary file5 Fig. 5 Post-ischemic recovery of LVDP in hearts given RBCs from healthy subjects (H RBC) that were incubated with (a) L-NAME (n=6), (b) ODQ (n=5) or (c) KT5823 (n=6). Post-ischemic recovery of LVDP in hearts (d) perfused with KT5823 (n=5) prior to administration of RBCs from healthy subjects. Post-ischemic LVDP is presented as percentage recovery from baseline. Data are presented as mean ± SD (JPG 2503 KB)

Supplementary file6 Representative immunofluorescence images showing P2Y13 receptor expression in RBCs from four different (a) patients with STEMI and (b) healthy subjects. Immunoreactivity was visualized using Alexa FluorTM 488 antibody (P2Y13, green) (JPG 2628 KB)

Supplementary file7 Post-ischemic recovery of LVDP in heart perfused (p) with vehicle (n=7) or PPADS (n=6) in KH buffer prior to administration of RBCs from STEMI patients (STEMI RBC). Post-ischemic LVDP is presented as percentage recovery from baseline. Data are presented as mean ± SD (JPG 659 KB)

Supplementary file8 Fig. 8 Post-ischemic recovery of LVDP in hearts given RBCs from healthy subjects (H RBC) that were incubated with (a) PPADS (n=5) or (b) MRS2211 (n=5). Post-ischemic LVDP is presented as percentage recovery from baseline. Data are presented as mean ± SD (JPG 1271 KB)

Supplementary file9 (DOCX 17 KB)

## Abbreviations

ATP: Adenosine triphosphate
eNOS: Endothelial nitric oxide synthase
LVDP: Left-ventricular developed pressure
LVEDP: Left-ventricular end-diastolic pressure
NO: Nitric oxide
PKG: Protein kinase G
RBCs: Red blood cells
sGC: Soluble guanylyl cyclase
STEMI: ST-elevation myocardial infarction
