# Supplementary figures and images for: Erythrocytes from patients with ST-elevation myocardial infarction induce cardioprotection through the purinergic P2Y13 receptor and nitric oxide signaling
Source: Basic Res Cardiol. 2022 Sep 16;117(1):46. doi: 10.1007/s00395-022-00953-4 (PMC9481504; doi:10.1007/s00395-022-00953-4)

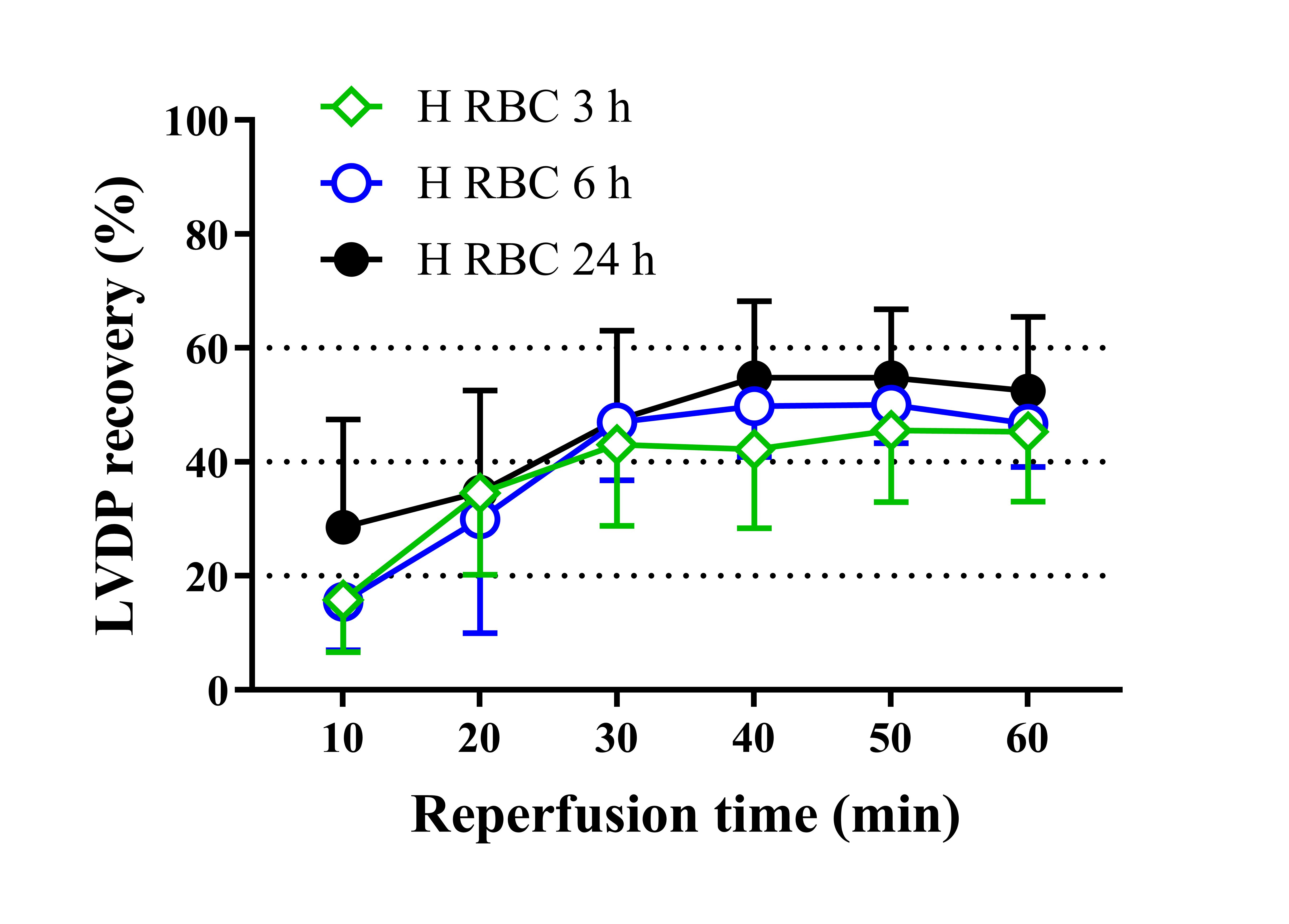

Supplement: Supplementary file 3 — Supplementary file3 Fig. 3 Effect of duration between RBCs collection and experimental determination of recovery of LVDP in isolated hearts subjected to global ischemia-reperfusion. Recovery of LVDP following administration of RBCs from healthy subjects (H RBC) after placing for 3 h (n=7), 6 h (n=8) or 24 h (n=7) at +4°C. Post-ischemic LVDP is presented as percentage recovery from baseline. Data are presented as mean ± SD. Statistical differences were analyzed with 2-way ANOVA including all time points (JPG 655 KB) [file 395_2022_953_MOESM3_ESM.jpg]

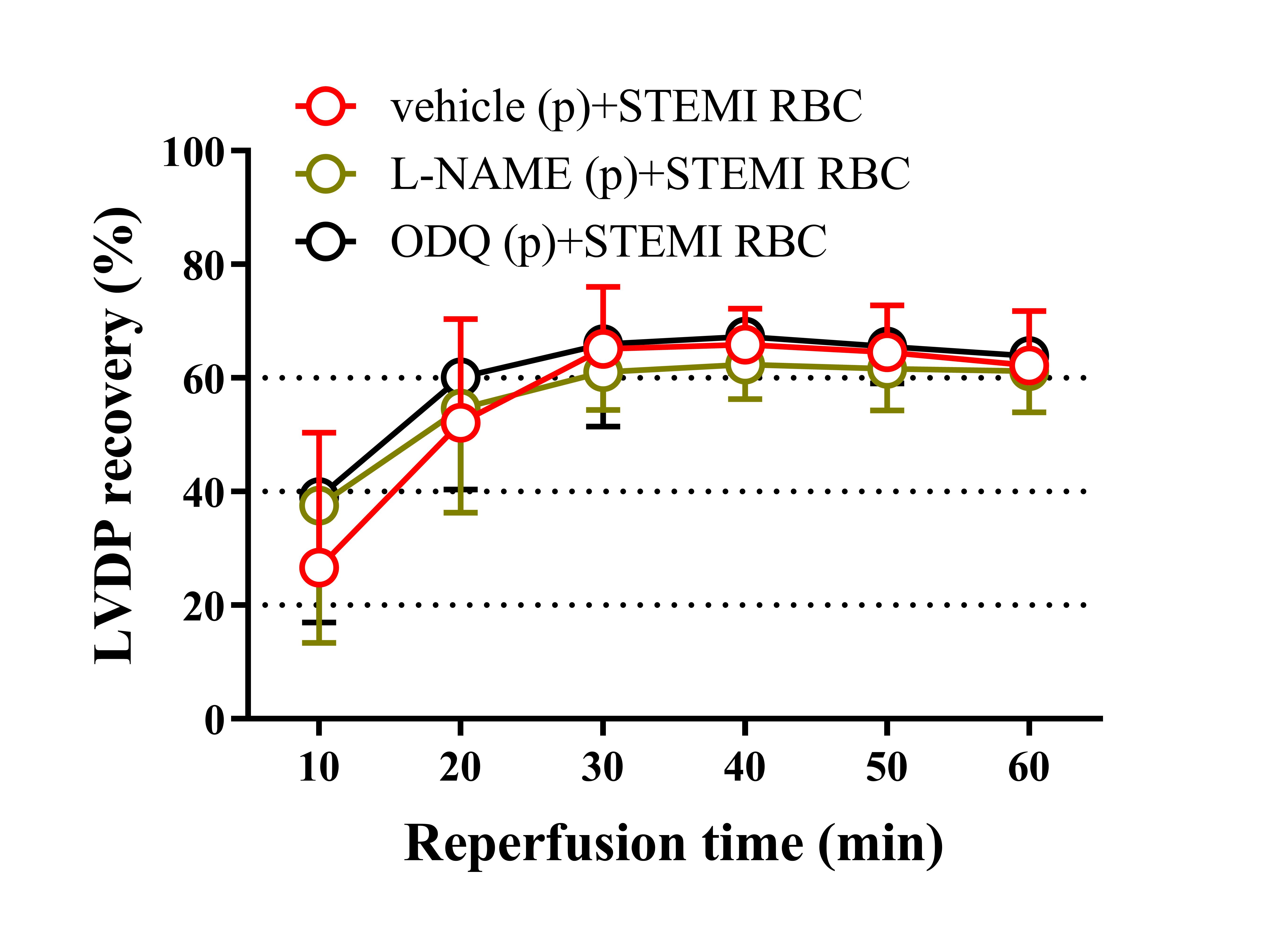

Supplement: Supplementary file 4 — Supplementary file4 Fig. 4 Post-ischemic recovery of LVDP in hearts perfused (p) with vehicle (n=7), L-NAME (n=6) or ODQ (n=6) in KH buffer prior to administration of RBCs from STEMI patients. Post-ischemic LVDP is presented as percentage recovery from baseline. Data are presented as mean ± SD (JPG 716 KB) [file 395_2022_953_MOESM4_ESM.jpg]

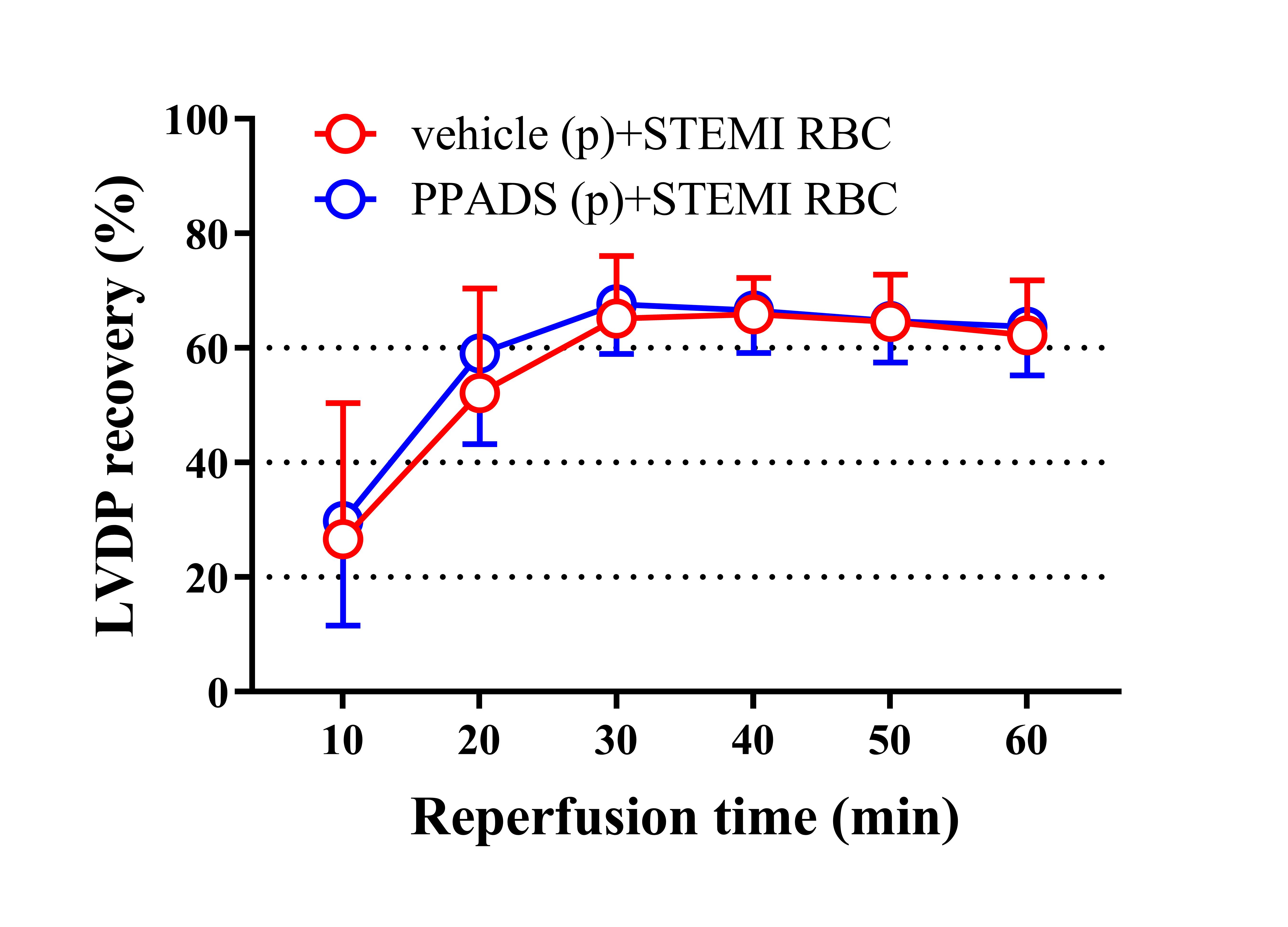

Supplement: Supplementary file 7 — Supplementary file7 Post-ischemic recovery of LVDP in heart perfused (p) with vehicle (n=7) or PPADS (n=6) in KH buffer prior to administration of RBCs from STEMI patients (STEMI RBC). Post-ischemic LVDP is presented as percentage recovery from baseline. Data are presented as mean ± SD (JPG 659 KB) [file 395_2022_953_MOESM7_ESM.jpg]

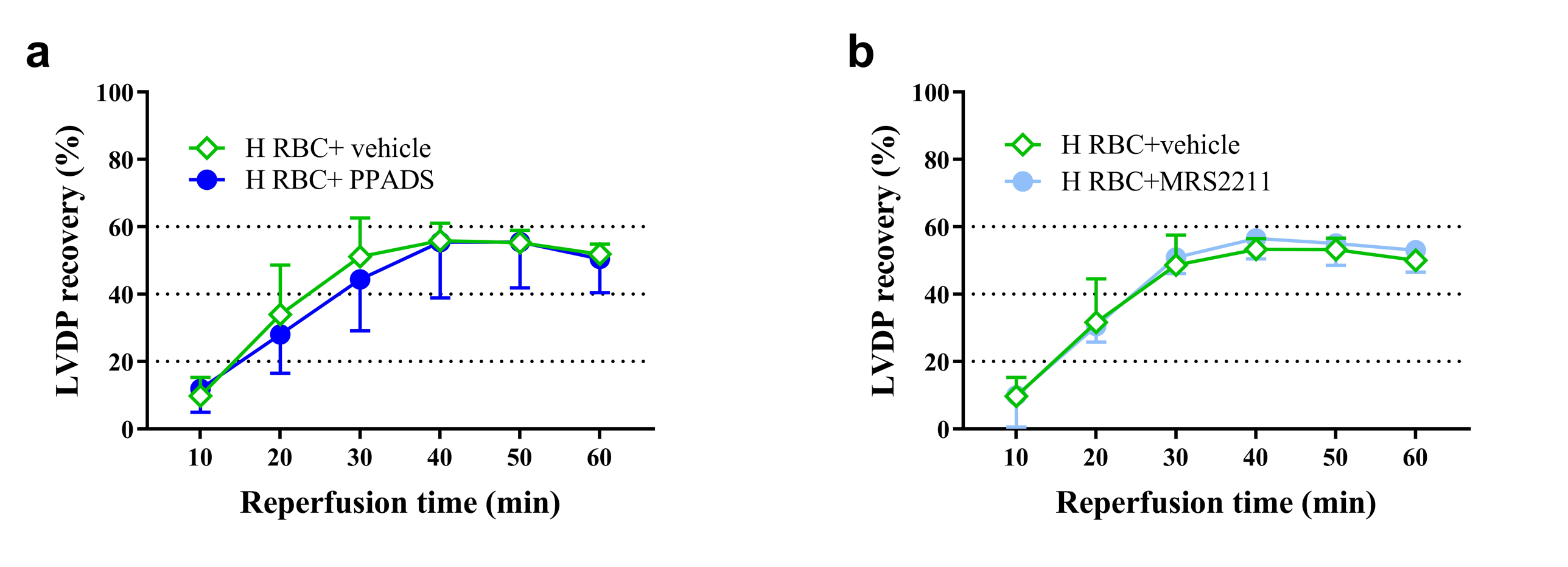

Supplement: Supplementary file 8 — Supplementary file8 Fig. 8 Post-ischemic recovery of LVDP in hearts given RBCs from healthy subjects (H RBC) that were incubated with (a) PPADS (n=5) or (b) MRS2211 (n=5). Post-ischemic LVDP is presented as percentage recovery from baseline. Data are presented as mean ± SD (JPG 1271 KB) [file 395_2022_953_MOESM8_ESM.jpg]
