# Supplementary material for: Erythrocytes from patients with ST-elevation myocardial infarction induce cardioprotection through the purinergic P2Y13 receptor and nitric oxide signaling
Source: Basic Res Cardiol. 2022 Sep 16;117(1):46. doi: 10.1007/s00395-022-00953-4 (PMC9481504; doi:10.1007/s00395-022-00953-4)
Supplement: Supplementary file 9 — Supplementary file9 (DOCX 17 KB) [file 395_2022_953_MOESM9_ESM.docx]

Supplementary Table 1. Baseline of isolated perfused rat hearts in Langendorff system.

| Group | n | LVSP  (mmHg) | LVED  P(mmHg) | LVDP  (mmHg) | dP/dt_max_ (mmHg/s) | HR  (beats/min) |
| --- | --- | --- | --- | --- | --- | --- |
| STEMI RBC+vehicle | 35 | 132±18 | 7±2 | 125±18 | 4369±785 | 286±29 |
| STEMI RBC+L-NAME | 6 | 146±7 | 6±3 | 139±8 | 4842±929 | 282±23 |
| STEMI RBC+ODQ | 8 | 150±19 | 7±2 | 143±19 | 4798±682 | 282±27 |
| STEMI RBC+PPADS | 7 | 148±19 | 7±3 | 141±18 | 5168±944 | 297±17 |
| STEMI RBC+8PT | 9 | 127±16 | 8±1 | 119±17 | 4148±1119 | 274±18 |
| STEMI RBC+MRS2211 | 6 | 138±10 | 5±1 | 133±10 | 4463±433 | 301±29 |
| KT5823(p)+STEMI RBC | 8 | 139±12 | 5±1 | 134±12 | 4297±1832 | 306±33 |
| vehicle(p)+STEMI RBC | 8 | 138±7 | 6±2 | 131±8 | 4788±854 | 297±33 |
| L-NAME(p)+STEMI RBC | 6 | 141±6 | 5±1 | 135±7 | 5481±817 | 279±26 |
| ODQ(p)+STEMI RBC | 6 | 139±9 | 6±1 | 133±9 | 5481±393 | 279±31 |
| PPADS(p)+STEMI RBC | 6 | 150±17 | 7±1 | 142±17 | 5425±1096 | 271±7 |
| H RBC+vehicle | 17 | 139±6 | 5±1 | 132±6 | 5008±851 | 301±26 |
| H RBC+mATP | 11 | 136±11 | 6±1 | 130±11 | 5304±702 | 298±37 |
| H RBC+mATP+ ODQ | 5 | 137±10 | 6±1 | 130±11 | 5623±614 | 301±15 |
| H RBC+mATP+ PPADS | 5 | 125±12 | 6±2 | 120±13 | 4820±832 | 298±25 |
| H RBC+mATP+ MRS2211 | 5 | 144±11 | 5±1 | 139±11 | 6020±493 | 307±21 |
| H RBC+L-NAME | 6 | 142±16 | 5±1 | 136±17 | 4795±802 | 290±25 |
| H RBC+ODQ | 5 | 140±11 | 5±2 | 134±9 | 5078±725 | 287±18 |
| H RBC+PPADS | 5 | 141±13 | 5±1 | 136±13 | 5306±755 | 314±38 |
| H RBC+MRS2211 | 5 | 141±21 | 6±2 | 135±20 | 4259±357 | 270±13 |
| H RBC 3h | 7 | 126±11 | 6±1 | 120±10 | 3549±692 | 283±20 |
| H RBC 6h | 8 | 119±12 | 6±1 | 112±13 | 3406±489 | 282±17 |
| H RBC 24h | 7 | 120±12 | 6±1 | 113±11 | 3595±691 | 281±16 |

Abbreviations: dP/dt_max_: maximal rate of rise in left ventricular pressure, H: heathy subject: HR: heart rate, LVEDP: left ventricular end diastolic pressure, LVDP: left ventricular developed pressure, LVSP: left ventricular systolic pressure, L-NAME: N^G^-nitro-L-arginine methyl ester, mATP: α-β-methylene ATP, ODQ: 1H- [1,2,4] oxadiazolo [4,3,-a] quinoxalin-1-one, PPADS: pyridoxal phosphate-6-azo(benzene-2,4-disulfonic acid) tetrasodium salt hydrate, 8PT: 8-phenyltheophylline, RBC: red blood cell, STEMI: ST elevation myocardial infarction. Data are presented as mean±SD.
